# High prevalence of lung cancer in a surgical cohort of lung cancer patients a decade after smoking cessation

**DOI:** 10.1186/1749-8090-6-19

**Published:** 2011-02-25

**Authors:** Cindy Mong, Edward B Garon, Clark Fuller, Ali Mahtabifard, James Mirocha, Zab Mosenifar, Robert McKenna

**Affiliations:** 1 Division of Hematology Oncology, UCLA David Geffen School of Medicine, 200 UCLA Medical Plaza, Suite 420 Los Angeles, CA 90024 USA; 2 Department of Cardiothoracic Surgery of Cedars Sinai Medical Center, 8635 West 3rd Street Suite 975 West Los Angeles, CA 90048 USA; 3 Department of Biostatistics Cedars Sinai Medical Center at 8700 Beverly Boulevard, Los Angeles, CA 90048 USA; 4 Division of Pulmonary Critical Care Medicine, Cedars Sinai Medical Center at 8700 Beverly Boulevard, Los Angeles, CA 90048 USA

## Abstract

**Background:**

This study was designed to assess the prevalence of smoking at time of lung cancer diagnosis in a surgical patient cohort referred for cardiothoracic surgery.

**Methods:**

Retrospective study of lung cancer patients (n = 626) referred to three cardiothoracic surgeons at a tertiary care medical center in Southern California from January 2006 to December 2008. Relationships among years of smoking cessation, smoking status, and tumor histology were analyzed with Chi-square tests.

**Results:**

Seventy-seven percent (482) had a smoking history while 11.3% (71) were current smokers. The length of smoking cessation to cancer diagnosis was <1 year for 56 (13.6%), 1-10 years for 110 (26.8%), 11-20 years for 87 (21.2%), 21-30 years for 66 (16.1%), 31-40 years for 44 (10.7%), 41-50 years for 40 (9.7%) and 51-60 years for 8 (1.9%). The mean cessation was 18.1 ± 15.7 years (n = 411 former smokers). Fifty-nine percent had stage 1 disease and 68.0% had adenocarcinoma. Squamous cell carcinoma was more prevalent in smokers (15.6% vs. 8.3%, p = 0.028); adenocarcinoma was more prevalent in never-smokers (79.9% versus 64.3%, p = 0.0004). The prevalence of adenocarcinoma varied inversely with pack year (p < 0.0001) and directly with years of smoking cessation (p = 0.0005).

**Conclusions:**

In a surgical lung cancer cohort, the majority of patients were smoking abstinent greater than one decade before the diagnosis of lung cancer.

## Background

Lung cancer is the leading cause of cancer death, accounting for 29% of total cancer deaths in the US and over 1.4 million deaths worldwide. In the US alone, lung cancer claimed the lives of 171,840 individuals in 2008 and an estimated 159,000 adults will die of lung cancer in 2009 as compared to a combined mortality of 118,000 adults from colorectal, breast, and prostate cancer [1]. Smoking plays an important causative role in lung cancer, first demonstrated in the British Physicians Study and validated through multiple epidemiologic studies thereafter [2-5]. Approximately 85% of lung cancer can be directly attributed to smoking and second hand smoke [2,3]. Moreover, the risk of developing lung cancer rises in a dose response relationship with total duration of smoking [2-5].

The definitive causative relationship between smoking and lung cancer has resulted in national efforts to reduce smoking. In the US, public health efforts have been successful. Smoking prevalence has decreased from 42.4% in 1965 to 17.9% in 2009 [6-10]. Yet, while there is an overall reduction, the residual effects of smoking on lung cancer risk remains most notable in former smokers and a significant proportion of lung cancer is now diagnosed in former smokers in the United States [11,12]. In a recent study of 5628 patients diagnosed with primary lung cancer, 89% had a smoking history and 49% were former smokers [13]. Former smokers disproportionately represent the majority of first time lung cancer patients.

Despite smoking cessation, lung cancer risk persists, as shown in the 12-year follow up of the Nurses Lung Health Study where the hazard ratio for lung cancer was 21.8 and 4.93 respectively for current and former smokers as compared to non-smokers[14]. For patients who are diagnosed with early stage lung cancer, surgical resection of the malignancy may be curative. This study was designed to evaluate the prevalence of smoking at the time of lung cancer diagnosis in a surgical cohort of lung cancer patients. This study's secondary aim was to examine relationships between smoking status, prevalence and years of smoking cessation, stage and histology of lung cancer.

## Methods

Retrospective analysis of 660 patients (January 2006-December 2008) with non-small cell and small cell lung cancer was performed at Cedar Sinai Medical Center. Thirty-four patients were excluded based on incomplete pathology reports. Patients were divided into subgroups based on stage of lung cancer (I, II, III, IV), and lung cancer pathology (adenocarcinoma, squamous cell, adenosquamous, small cell, large cell, bronchoalveolar carcinoma, and undifferentiated carcinoma) based on site of primary tumor. Smoking status was self-reported by the patient. Patients described their own smoking status as either non-smoker or history of smoking. For patients who had a history of smoking, the choices for describing smoking history were packs per day, and number of years smoked. No investigation was performed to verify the accuracy of the patient's self-reported smoking history. Analysis was performed using the data set generated from intake history questionnaire and pathology reports. The protocol was approved by the institutional review board of Cedar Sinai Medical Center (IRB Pro00019964) and was carried out in accordance with its ethical and legal requirements.

### Statistical Analysis

Numerical variables were summarized as mean ± standard deviation or median (interquartile range [IQR]). Categorical variables were summarized as frequencies and percents. The smoking cessation length variable was grouped into ordinal levels by 10-year increments: 0-10 years, 11-20 years, 21-30 years, 31-40 years, 41-50 years and 51-60 years. The pack year (PY) variable was grouped into ordinal levels by increments: 0, 1-20, 21-50, 51-100, and 100+ pack years. The adenocarcinoma and non-adenocarcinoma histology variables were grouped as categorical variables. The squamous cell carcinoma and non-squamous cell carcinoma histology were grouped as categorical variables.

Relationships between categorical variables were assessed by Chi-Square or Fisher exact tests. The Cochran-Armitage trend test (1-sided) was used to assess the relationship between the percent of patients with Adenocarcinoma and PY and the relationship between the percent of patients with Adenocarcinoma and length of smoking cessation. Relationships between ordinal variables were assessed by Spearman correlation. The 5% significance level was used throughout. Statistical calculations were performed using SAS version 9.1 (SAS Institute, Cary, NC). Figures were created using Microsoft Excel (Microsoft Excel for Mac 2004, Version 11.5.8).

## Results

**Figure 1:**
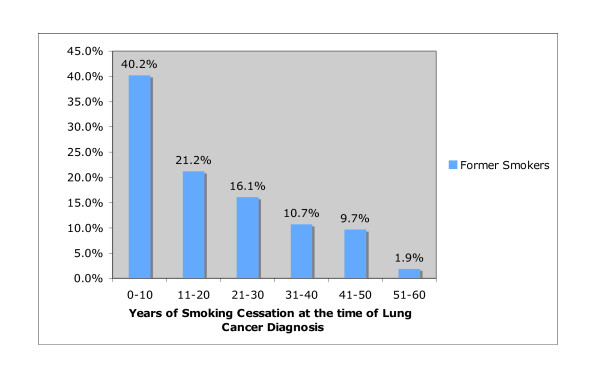
**Years of Smoking Cessation to Lung Cancer Diagnosis versus Percent of Former Smokers, n = 411**. Sixty percent of patients in a surgical lung cancer cohort with a smoking history had stopped smoking 11-60 years (mean 18.1 ± 15.7, median 16, IQR 3 to 30) before lung cancer diagnosis.

Six-hundred twenty six complete patient records were identified and included in analysis. Of these 626 patients, 321 (51.3%) were male and 305 (48.7%) were female. The mean age was 70.1 ± 10.9 years. (Table 1) Seventy-seven percent had a smoking history but only 11.3% of patients were current smokers. For former smokers (n = 411), the length of smoking cessation to cancer diagnosis was <1 year for 56 (13.6%), 1-10 years for 110 (26.8%), 11-20 years for 87 (21.2%), 21-30 years for 66 (16.1%), 31-40 years for 44 (10.7%), 41-50 years for 40 (9.7%) and 51-60 years for 8 (1.9%). (Figure 1) Of patients with a smoking history, 14.7% were ongoing smokers. (Table 1) Among patients with a smoking history, patients had quit smoking an average of 18.1 ± 15.7 years (median 16, IQR 3 to 30) before they were diagnosed with lung cancer. Of patients with a smoking history, 117 (18.7%) smoked 1-20 packs per year, 227 (33.0%) had smoked 21-50 pack years, and 131 (20.9%) had smoked 51-100 pack years. Three hundred sixty-eight (58.8%) patients were found to have stage 1 disease, 96 (15.4%) had stage 2 disease, 149 (23.8%) had stage 3 disease and 13 (2.1%) had stage 4 disease. (Table 1) Adenocarcinoma was the most common histology, with an overall prevalence of 68%. The non-adenocarcinoma lung cancer types included 87 (13.9%) squamous cell carcinoma, 34 (5.4%) adenosquamous cell carcinoma, 32 (5.1%) bronchoalveolar cell carcinoma, 24 (3.8%) large cell carcinoma, 14 (2.2%) small cell carcinoma, and 10 (1.6%) undifferentiated non-small cell lung cancer.

**Table 1:** Patient Characteristics (n = 626)

Demographics	Number	%
**Gender**		

Male	321	51.3

Female	305	48.7

Age (Mean ± SD)	70.1 ± 10.9	

Age (Median, Range)	71, 22-97	

Age ≤ 70	304	48.6

Age > 70	322	51.4

**Pathologic Stage**		

1	368	58.8

2	96	15.3

3	149	23.8

4	13	2.1

**Years of Smoking Cessation for Former Smokers: Mean ± SD**	18.1 ± 15.7	

**Years of Smoking Cessation for Former Smokers: Median, IQR**	16, 3-30	

Less than 1 year	56	13.6

1-10 years	110	26.8

11-20 years	87	21.2

21-30 years	66	16.1

31-40 years	44	10.7

41-50 years	40	9.7

51-60 years	8	1.9

**Smoking Status**		

Current Smokers	71	11.3

Former Smokers	411	65.7

Never-smokers	144	23.0

**Pack Year (PY)**		

0	144	23.0

1-20	117	18.7

21-50	207	33.1

51-100	131	20.9

≥ 100	27	4.3

**Tumor Histology**		

Adenocarcinoma	425	67.9

Non-adenocarcinoma	201	32.1

**Detailed Tumor Histology**		

Adenocarcinoma (A)	425	67.9

Adenosquamous (ASQ)	34	5.4

Squamous (SQ)	87	13.9

Bronchoalveolar (BAC)	32	5.1

Large Cell (L)	24	3.8

Small Cell (S)	14	2.2

Undifferentiated (U)	10	1.6

**Figure 2:**
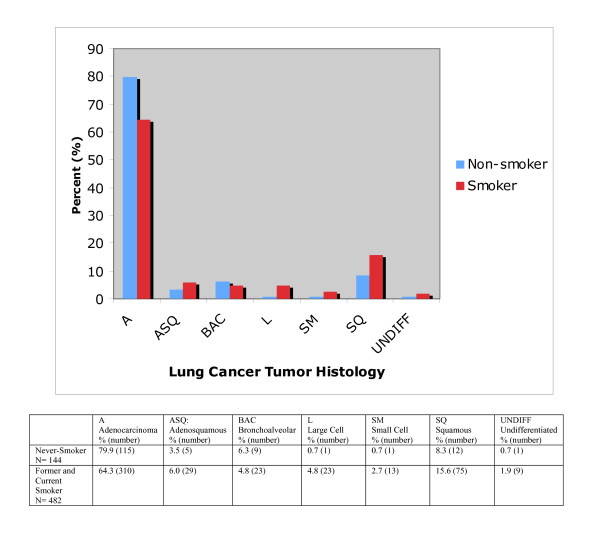
**Lung Cancer Histology by Smoking Status**. Lung Cancer histology differs by smoking status (p = 0.007). The percent of lung cancer patients with adenocarcinoma was lower in current and former smokers than in never-smokers (64.3% versus 79.9%, p = 0.0004), while the percent with squamous cell carcinoma was higher in smokers than in never-smokers (15.5% versus 8.3%, p = 0.028).

The histology of lung cancer differed across smoking status. (p = 0.007, Figure 2) Post hoc tests showed that the percent of lung cancer patients with adenocarcinoma was lower in smokers than in never-smokers (64.3% versus 79.9%, p = 0.0004). In contrast, the percent with squamous cell carcinoma was higher in smokers than in never-smokers (15.5% versus 8.3%, p = 0.028). (Figure 2)

**Figure 3:**
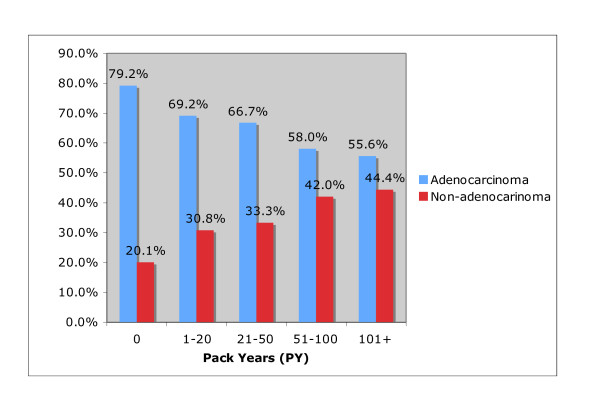
**Pack Years (PY) versus Lung Cancer Tumor Histology, n = 626**. The percent of lung cancer patients with adenocarcinoma decreases with increasing Pack Years (PY), p < 0.0001.

**Figure 4:**
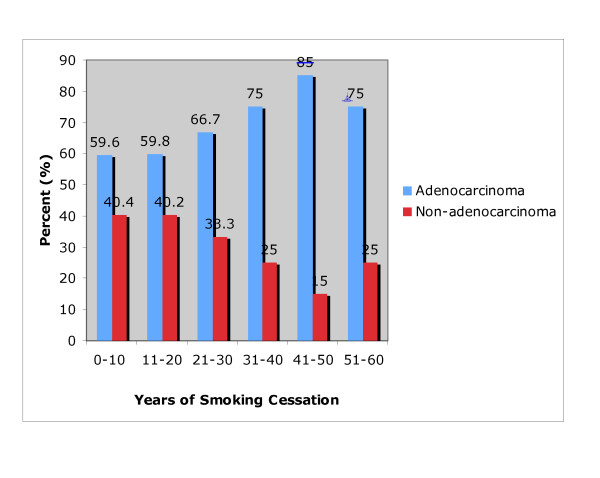
**Relationship between Years of Smoking Cessation and Tumor Histology n = 411 (former smokers)**. The percent of patients with Adenocarcinoma increases with years of smoking cessation, p = 0.0005.

Among persistent smokers, the percent of patients with adenocarcinoma varied inversely with pack years. As pack years (PY) increased, the percentage of adenocarcinoma decreased (p < 0.0001). (Figure 3) The likelihood of having adenocarcinoma varied directly with years of smoking cessation. (p = 0.0005) (Figure 4)

## Discussion

Smoking plays an important causative role in the pathogenesis of lung cancer [2-5]. Lung cancer patients may face stigmatization for having smoked in the past or present, and are widely believed to be ongoing smokers who resist cessation [15]. In this cohort of surgical lung cancer patients, the prevalence of lung cancer was high in those patients who quit smoking over one or more decades before developing lung cancer. In this cohort, only 11.3% of all patients and 14.7% of patients with a smoking history were current smokers. Consistent with prior studies, we found that, in our cohort, adenocarcinoma was less prevalent in patients with a heavy smoking history [16,17].

Our study supports that smoking plays an important causative role in lung cancer pathogenesis, years after smoking cessation. Studies cite the "quitting ill effect" or the excessive lung cancer risk experienced by former smokers 5 years directly following smoking cessation. Garfinkel and Stellman observed that while smokers often quit as a result of symptoms or a life threatening condition, the risk of lung cancer immediately following cessation is often greater than that for smokers who continue to smoke [18,19]." Even though these patients had quit smoking, they may be considered ongoing smokers as they often quit smoking as a direct result of lung cancer symptoms such as shortness of breath, cough, and hemoptysis. Our study illustrates that having a long-term smoking history itself, whether the patient is a persistent smoker or not, predisposes the patient to increased cancer risk.

While the surgical cohort of lung cancer patients had generally stopped smoking decades before, tobacco's carcinogenic effects persisted. (Figure 1) Case control and cohort studies show a 50% decrease in the risk of lung cancer within first 15 years of abstinence, but the risk never drops to that of non-smokers [20]. A prospective cohort study of 41,836 women aged 55 to 69 years showed that the relative risk remained 6.6 for all former smokers 30 years after smoking abstinence[21]. Furthermore, studies suggest that excess lung cancer risk persists beyond 10 to 15 years of smoking abstinence [22]. Sixty percent of our cohort developed lung cancer despite stopping smoking over one decade ago.

The major limitation of our study is the selection bias for lung cancer patients who were referred to cardiothoracic surgeons in a tertiary care medical center. Our surgical lung cancer cohort may represent an atypical lung cancer cohort group. The vast majority of our study patients had early stage disease and was asymptomatic at the time of diagnosis. In our cohort, 58.8% of patients were diagnosed stage 1, 15.3% were diagnosed with stage 2 disease, while 25.9% patients presented with stage 3 and 4 disease. In contrast, in the general population, more than half of lung cancer patients are diagnosed at an advanced stage, and they are more likely to experience shortness of breath, cough, and hemoptysis and/or weight loss and fatigue. From 1999-2006, the National Cancer Institute showed that only 16% of lung cancer was diagnosed at the early stage, while 25% were diagnosed after they had spread regionally beyond the primary site to lymph nodes and 51% were diagnosed with distant metastases [23].

Surgically referred lung cancer patients may be more likely to be former smokers as compared to patients who are diagnosed with advanced stage disease. We noted that, in the patient cohort referred for cardiothoracic surgery, only 11.3% current smokers. In contrast, a sectional study of all lung cancer patients from 1986 to 1990 at M.D. Anderson Cancer Center found that 47.8% of lung cancer patients were current smokers[24]. The MD Anderson study represented all lung cancer patients referred for medical and surgical management of lung cancer. Our findings may not generalizable to the overall lung cancer population.

Extrapolating patterns from our cohort to the general population is also limited by our study's reliance on retrospective data collection and the referral center's location in Southern California. First, data collection was dependent on patient reporting smoking history. Given the stigmatization of smoking in the medical community, patients may be more likely to underestimate their pack year smoking history [25]. Furthermore, patient selection bias may also derive from the study center's location in Southern California. This medical center will typically see residents of Southern or Northern California. California was one of the first states to ban on smoking in all enclosed workspaces (1995), and subsequently enacted strict anti-smoking laws in the majority of public spaces [26]. Thus, patients in California may have had disproportionately higher rates of smoking cessation following anti-smoking laws implementation as compared to other states [27]. In the first decade after a comprehensive tobacco control program was implemented in California (1988), there was a 6% reduction in lung cancer incidence and 11,000 lung cancer cases were avoided [28]. Our patient cohort may be more likely than patients in other states to be former smokers as a result of environmental pressures. Recent studies showed that smoke-free workplaces are associated with reductions in prevalence of smoking of 3.8% and 3.1 fewer cigarettes smoked per day per continuing smoker [29].

## Conclusions

In conclusion, there is a low prevalence of ongoing smoking in our lung cancer surgical cohort; and the majority of these patients were smoking abstinent at least one or more decades. The median length of smoking cessation was 16 (3 to 30) years, and the mean length of smoking cessation was18.1 ± 15.7 years (Figure 1) As more states adopt stringent tobacco control programs like California, we may expect a proportional increase in the prevalence of smoking cessation, a lengthened period of smoking cessation and an increase in the proportion of early stage lung cancers at time of diagnosis. This prediction is supported by data gathered following enactment of California's Tobacco Control programs. Following the passage of the 1988 California Comprehensive Tobacco Control Program, California experienced a 18% decrease in lung cancer incidence from 1990-94 to 1995-99 [30]. From 1988-99, lung cancer rates declined 19.5% according to the California Department of Health Services. In contrast, states with lenient tobacco programs noted a rise in lung cancer rates while states with stringent tobacco control programs experienced an accelerated decline in lung cancer rates [31]. Specifically, the California Tobacco Control program was associated with a significantly greater rate of decline in the age-adjusted incidence rate for lung cancer as compared to eight other sites across the nation. ^32 ^Support for this trend was shown as California's 1999 rate of lung cancer was 10.4% lower than the national incidence rate of lung cancer [32]. Our study may give us a glimpse into the future of lung cancer and its changing demographic trends in California and the US.

Despite strides in smoking prevention and environmental pressures to discourage smoking, it is clear that lung cancer will continue to be a major threat to public health. While rates of death from breast and prostate have leveled off, death from lung and bronchus cancer has only begun to plateau from their rapidly rising mortality rate. Understanding the patterns of smoking history, tumor stage/histology, and years of smoking cessation in our surgical lung cancer cohort may allow the clinician to better counsel our patients to optimize their cancer management and promote cancer prevention. A substantial risk of developing lung cancer persists in former smokers many years following smoking cessation. If screening for lung cancer is recommended in the future, it should be continued for decades after smoking cessation.

## Competing interests

The authors declare that they have no competing interests.

## Authors' contributions

CM conceived of the study, performed data acquisition, interpretation and analysis and interpretation of data, drafted the manuscript and created figures and tables. EG conceived of the study design with CM, and performed data analysis, data interpretation, paper revision, and manuscript revision. JM conducted statistical analysis, contributed to paper revision and manuscript draft, and tables and figures design. CF contributed to data, and paper revision. RM was instrumental in data collection, and paper revision and participated in its design and coordination. ZM was instrumental in paper revision. All authors read and approved the final manuscript.
